# Sectional Anatomy with Micro-Computed Tomography and Magnetic Resonance Imaging Correlation of the Middle and Caudal Abdominal Regions in the Syrian Hamster (*Mesocricetus auratus*)

**DOI:** 10.3390/ani15091315

**Published:** 2025-05-01

**Authors:** Nima Mohammadzadeh, Jamal Nourinezhad, Abdolvahed Moarabi, Maciej Janeczek

**Affiliations:** 1 Graduated D.V. M. Student of Faculty of Veterinary Medicine, Shahid Chamran University of Ahvaz, Ahvaz 61357-83151, Iran; nima.m1999@icloud.com; 2 Division of Anatomy and Embryology, Department of Basic Sciences, Faculty of Veterinary Medicine, Shahid Chamran University of Ahvaz, Ahvaz 61357-83151, Iran; 3 Division of Radiology, Department of Clinical Sciences, Faculty of Veterinary Medicine, Shahid Chamran University of Ahvaz, Ahvaz 61357-83151, Iran; a.moarabi@scu.ac.ir; 4 Division of Animal Anatomy, Department of Biostructure and Animal Physiology, Faculty of Veterinary Medicine, Wroclaw University of Environmental and Life Sciences, 50-375 Wrocław, Poland; maciej.janeczek@upwr.edu.pl

**Keywords:** abdominal region, cross-sectional imaging, gross anatomy, planar anatomy, rodent

## Abstract

The abdomen is a key region in small animal veterinary practice, with the middle and caudal sections housing various organ systems that are susceptible to dysfunction, necessitating medical intervention or surgery. Sectional imaging techniques like CT and MRI are commonly used in small mammals, but no studies have focused on rodent abdomen. This study aimed to correlate micro-CT and MRI images of the middle and caudal abdominal regions with corresponding anatomical sections in Syrian hamsters (SHs), which are popular pets and experimental models. Ten healthy male SHs were used, and anatomical structures from frozen sections were compared with corresponding MCT and MRI images. Clinically relevant structures identified in anatomical sections were discernible on MCT and MRI scans. The key findings include the presence of glandular and non-glandular stomachs, the stomach and cecum primarily located on the left side, the absence of ampulla coli, sacculus rotundus, and cecal appendix, and sacculation of the colon, as well as the jejunum, mainly on the right side. The vesicular, coagulating, and prostate glands were also present, and the right kidney did not extend to the last thoracic vertebra. The results were similar to abdominal anatomical and radiologic studies in rats, mice, and guinea pigs, regardless of the rat’s and mice’s sacculated cecum and the guinea pig’s glandular stomach. However, significant differences were observed compared to the rabbit abdomen’s sectional anatomy and CT findings. This study highlights the diagnostic value of MCT and MRI in SHs and provides a valuable reference for interpreting cross-sectional abdominal images in SHs.

## Simple Summary

Although Syrian hamsters are the most common pet and experimental hamster species, reports on the abdominal sectional anatomy and imaging in this species are lacking. Advanced imaging diagnostic techniques, such as micro-computed tomography and magnetic resonance imaging with anatomical sections, were used to evaluate the middle and caudal abdominal regions of the Syrian hamster. The images and data presented in this study could be considered a valuable atlas for anatomists, radiologists, clinicians, surgeons, and researchers.

## 1. Introduction

The abdomen extends from the diaphragm to the pelvis and has basically three regions: cranial abdominal, middle, and caudal abdominal [1]. The middle and caudal regions contain a wide range of organ systems, with plenty of opportunities for these to go wrong and require treatment and surgery.

The abdomen is one of the areas most evaluated in small animal veterinary practice [2], and the acquisition of abdominal radiographs is one of the most common radiographic examinations [3]. In survey radiographs, intraabdominal organs are typically present with soft tissue or fluid opacity [4]. This results in limitations in accurately differentiating specific organs due to similar X-ray absorption levels. In small exotic mammals, the reduced size of abdominal arrangements and poor organ contrast further complicate radiographic interpretation [5].

However, cross-sectional imaging modalities, including computed tomography (CT) and magnetic resonance imaging (MRI), are increasingly being used for the diagnostic imaging of small exotic mammals as they are available within both private practice and academic sectors [6] and offer significant advantages over conventional radiography, mainly due to their tomographic capabilities and superior contrast resolution [3] and can be used to depict the position and relations of arrangements without the problem of superimposition [7]. In small animals, CT is used extensively to evaluate the abdomen, but its resolution may be insufficient for the detailed assessment of smaller abdominal arrangements [8,9]. To overcome the limitation in the abdomen, micro-CT has emerged as a valuable tool, offering higher spatial resolution, reduced scanning times, and cost-effectiveness, and is generally used to provide high-resolution anatomic images of small animals [10,11]. These attributes make micro-CT particularly useful in research focused on abdominal organs [10,12,13], as well as specific diagnostic and clinical abdominal applications in small animals [14].

Parallel to this, MRI is an essential imaging modality for abdominal diagnostics in small exotic mammals [6,15], offering superior soft tissue contrast and detailed anatomical information [16]. This capability is particularly important in the abdominal region for staging malignancies, monitoring therapeutic efficacy [17,18], and identifying gastrointestinal pathologies [19]. MRI’s ability to distinguish between different tissue types and its utility in both clinical [15] and research [20] contexts make it an indispensable complement to CT for comprehensive abdominal imaging in small mammals such as rodents and rabbits [6].

On the other hand, ultrasound imaging provides partial views of anatomy in each cross-slice, and it is restricted by its inability to penetrate gas and mineralized tissues, which further limits the observable range within the abdomen [21].

To accurately evaluate and interpret CT/micro-CT or MRI images of the abdomen, it is essential to know the sectional anatomy of a clinically normal abdomen [22]. Macroscopic anatomy knowledge of the abdomen is a prerequisite and the first step toward understanding cross-sectional anatomy [2].

Literature reviews show that few comparative studies between the CT and/or MRI and cross-sectional anatomy of the abdomen have been published in small mammals, including rabbits [23], cats [21], and dogs [24]. Only a few CT and MR images of the normal abdomen are also presented in rabbits and laboratory rats [25], as well as the kidney and liver in Chinchilla [26,27], without correlation with anatomical slices. Moreover, the CT of the abdominal organs, including gastrointestinal tracts [8,28], the spleen [29], the urinary tract [30], the prostate [31], and vascular glands [32] of rabbits, as well as the digestive tract of guinea pigs [28], without comparison with gross slices are reported. MR investigation of the liver [33], prostate glands [34], bulbourethral glands [35], kidneys, ureters, and urinary bladders [36] without comparison with gross slices is also documented in rabbits. Nevertheless, correlative serial abdominal CT/micro-CT/and/or MR image references with gross anatomic slices currently seem to be unavailable for rodents.

Hamsters are very popular as pet animals [37], and the Syrian hamster is the most common pet hamster. Approximately 90% of hamsters used in research are of Syrian origin [38]. For example, they are suitable models for evaluating serial bile duct changes [18] and pancreatic ductal adenocarcinomas [39]. Clinically, they are prone to various health issues, including gastrointestinal diseases [40,41], diabesity [42], abdominal masses, traumatic injuries [41], renal failure [43], and mammary [44], pancreatic [45], and gastrointestinal [46] tumors. Therefore, the primary objectives of this investigation were (1) to document for the first time the colorful descriptions of the morpho-topography of clinically major abdominal arrangements in male Syrian hamsters, (2) to provide sequential gross slices of the abdomen, (3) to describe clinically relevant abdominal micro-CT and MRI anatomy, and (4) to correlate cadaveric slices and micro-CT and MRI scans. This study is consistent with our reports concerning the thoracic anatomy of Syrian hamsters [47,48,49] and the correlative imaging anatomy of the Syrian hamster head [50] and thorax [51].

## 2. Materials and Methods

### 2.1. Examined Animals

Ten healthy adult male intact Syrian hamsters (mean ± SD weight: 104.83 ± 2 g; 69 days) were used in this investigation. The age of hamsters was determined based on body weight, as reported by Gad [52]. The hamsters were found to be healthy based on individual history and clinical examination, and whole-body radiologic and ultrasonographic (Z60 Vet, Shenzhen Mindray Animal Medical Technology Co., Shenzhen, China) analyses were obtained on each subject. The hamsters, sourced from the Razi Vaccine and Serum Research Institute and the Center for Comparative and Experimental Medicine at Shiraz University of Medical Sciences, were kept in an air-conditioned environment with free access to water and standard commercial chow on a 12 h light/dark cycle. No fasting was required before radiographic, micro-CT, and MRI examinations [53]. All procedures were approved by the Local Ethical Committee for Animal Use in Experiments (Approval Code: IR.SCU.REC.1403.110). Prior to imaging, hamsters were anesthetized intraperitoneally with a mixture of 5 mL ketamine (Bremer Pharma GMBH, Warburg, Germany), 1 mL acepromazine (Alfasan, Woerden, The Netherlands), and 2.5 mL xylazine (Alfasan, Woerden, The Netherlands), as described in the guidelines by McGill University (https://www.mcgill.ca/research/files/research/112-hamster_anesthesia_-_jan_2021_v2.pdf, accessed on 1 January 2021).

As a preliminary step, abdominal imaging was conducted using a 64-slice CT scanner (Siemens, Somatom Emotion, Erlangen, Germany) and a 1.5 Tesla MRI system (Phillips, Ingenia, Eindhoven, The Netherlands).

### 2.2. Radiological Procedures

Lateral and ventrodorsal abdominal radiographs were taken using an X-ray unit (300 MA, Toshiba, Otawara, Japan) with positioning techniques adapted from Silverman and Tell [25].

### 2.3. Micro-CT Procedures

Abdominal evaluations were performed in ventral recumbency using an in vivo X-ray micro-CT scanner (LOTUS in Vivo, Behin Negareh Co., Tehran, Iran) at the Preclinical Core Facility based at Tehran University of Medical Sciences. Positioning followed the methods described by Capello and Lennox [54]. Imaging settings included an X-ray tube voltage of 80 kV, a current of 100 µA, and a frame exposure time of 0.25 s at 1.3× magnification. The scanning took 30 min, and the image slices were reconstructed at 30-micrometer thickness. The imaging protocol was managed by LOTUS-in vivo-ACQ software, version 1.2 and 3D data were reconstructed using the standard Feldkamp–Davis–Kress (FDK) algorithm, producing DICOM images in transverse, sagittal, and dorsal planes. Window width and level adjustments optimized imaging for thoracic arrangements.

### 2.4. MRI Procedures

The abdomen was evaluated by MRI on a 3 Tesla clinical scanner (Siemens, MAGNETOM Prisma, Erlangen, Germany) using a 20-channel head coil. Turbo spin-echo (SE) T1-weighted (T1W) and T2-weighted (T2W) images were acquired in sagittal, dorsal, and transverse planes. T2-weighted images had parameters of a TE of 3.46 ms, a TR of 6110.0 ms, a voxel size of 0.4 × 0.4 mm, a slice thickness of 1.2 mm, and a field of view (FOV) of 64 × 82 mm. For sagittal images, the TE was 103 ms, TR 4480 ms, slice thickness 1.6 mm, and FOV 70 × 90 mm. To optimize image quality and reduce the animal’s health risks associated with prolonged scan times and anesthesia in a clinical setting [15,55], such slice thicknesses [56] and voxel sizes [57] were selected. The Bee DICOM Viewer 2.5.1 (416, Beijing, China) was used for image evaluation.

### 2.5. Anatomical Examinations

A total of eight Syrian hamsters were euthanized: two for topographic anatomy and six for anatomical sectioning.

#### 2.5.1. Topographic Anatomy

Following imaging, two Syrian hamsters were euthanized according to AVMA guidelines [58] and then dissected and photographed using a Samsung Galaxy S24 Ultra (Samsung Galaxy, Suwon, Korea).

#### 2.5.2. Anatomical Sectioning

Six euthanized Syrian hamsters were frozen at −70 °C in ventral recumbency and then sliced using an electronic bandsaw. Approximately five millimeter-thick slices were obtained along the transverse, sagittal, and dorsal planes and photographed. Identifiable anatomical arrangements were labeled based on available references [59,60] and in accordance with the Nomina Anatomica Veterinaria [61]. Correlations between micro-CT, MRI, and cadaver slices were drawn to label recognizable arrangements across modalities. However, some arrangements visible in the anatomical slices were undetectable on MRI and/or micro-CT images.

## 3. Results

**Figure 1:**
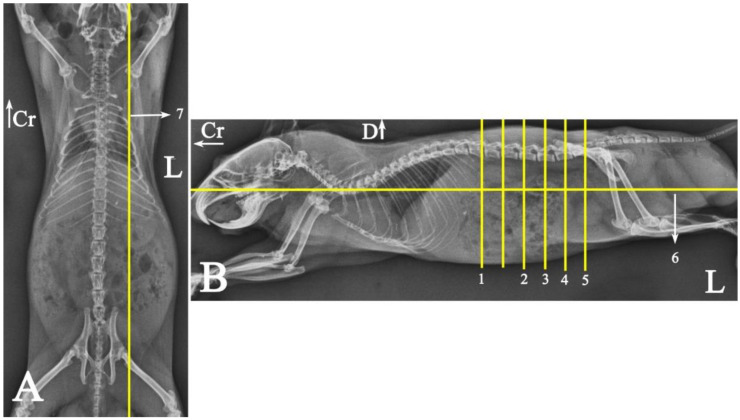
Radiographs of a male Syria hamster’s abdomen: (VD) ventrodorsal (**A**) and (L) left lateral (**B**) view. Yellow lines indicate the approximate dispositions of anatomical slices and sectional imaging, with numbers corresponding to their respective images. The unnumbered line is the slice that has not been described due to its arrangement similarity to other slices: Cr: cranial, D: dorsal, L: left side of the body.

**Figure 2:**
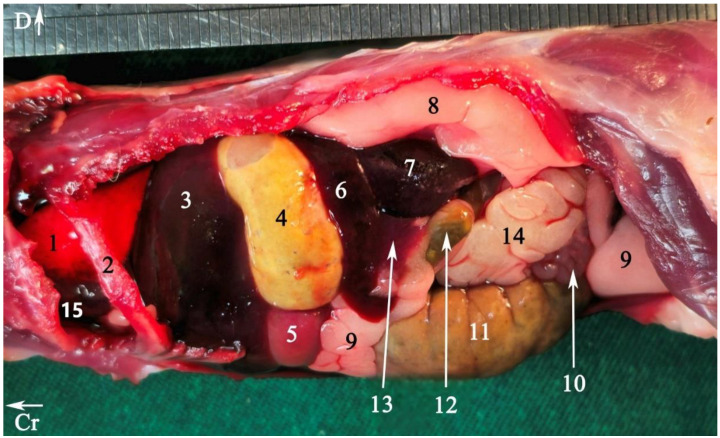
Abdominal viscera of a fresh cadaver of male Syrian hamster *in situ*: left lateral view. To provide greater details of the arrangements, part of the diaphragm and costal arch have been removed. For better visualization of the kidney, the perinephric fat has been retracted dorsally. (1) Lung, (2) rib number 7, (3) liver, (4) non-glandular part of the stomach, (5) glandular part of the stomach, (6) spleen, (7) left kidney, (8) perirenal fat, (9) adipose tissue, (10) coagulating gland, (11) cecum, (12) descending colon, (13) pancreas, (14) seminal vesicle gland; (15) heart. Cr: cranial, D: dorsal.

**Figure 3:**
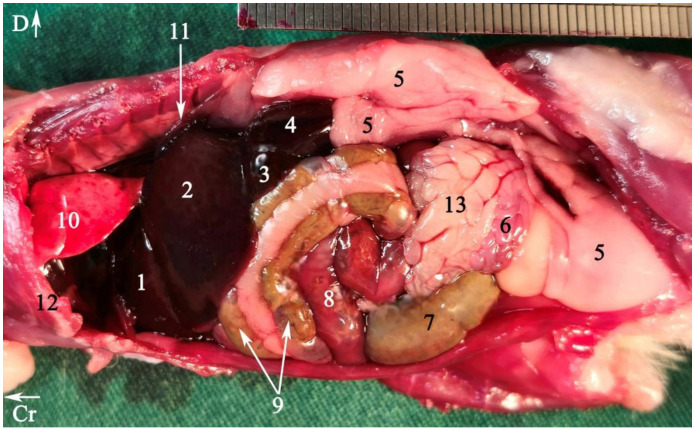
Abdominal viscera of a fresh cadaver of male Syrian hamster *in situ*: right lateral view. To provide greater details of the arrangements, part of the diaphragm and rib arch have been removed. For better visualization of the kidney, the perirenal fat has been displaced dorsally. (1) Right medial lobe of the liver, (2) right lateral lobe of the liver, (3) caudate lobe of the liver, (4) right kidney, (5) adipose tissue, (6) coagulating gland, (7) cecum, (8) jejunum, (9) ascending colon, (10) lung, (11) diaphragm, (12) rib number 7, (13) seminal vesicle gland. Cr: cranial, D: dorsal.

**Figure 4:**
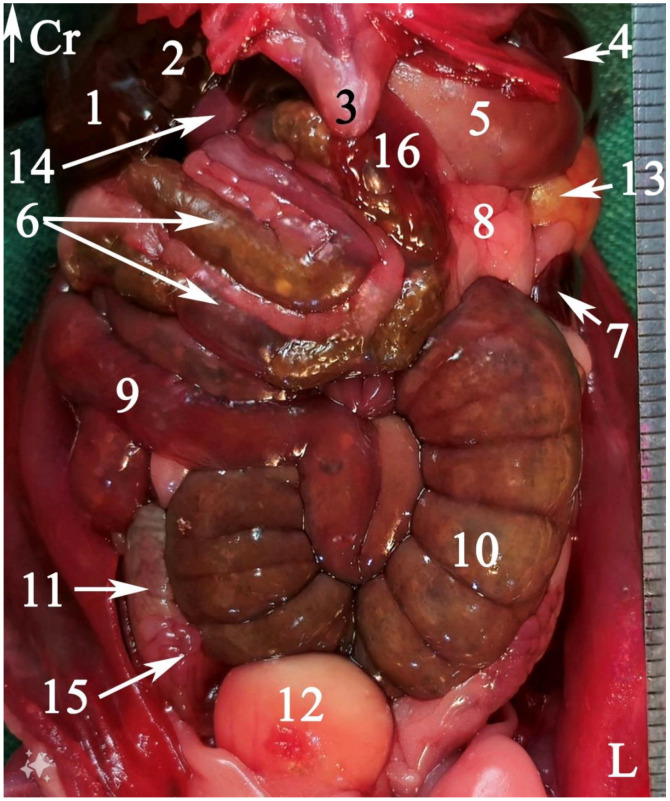
Abdominal viscera of a fresh cadaver of a male Syrian hamster *in situ*: ventral view. (1) Right lateral lobe of the liver, (2) right medial lobe of the liver, (3) xiphoid cartilage, (4) left lateral lobe of the liver, (5) glandular part of the stomach, (6) ascending colon, (7) spleen, (8) adipose tissue, (9) jejunum, (10) cecum, (11) seminal vesicle gland, (12) bladder, (13) non-glandular part of the stomach, (14) duodenum, (15) coagulating gland, (16) pancreas. Cr: cranial, L: left side of the body.

**Figure 5:**
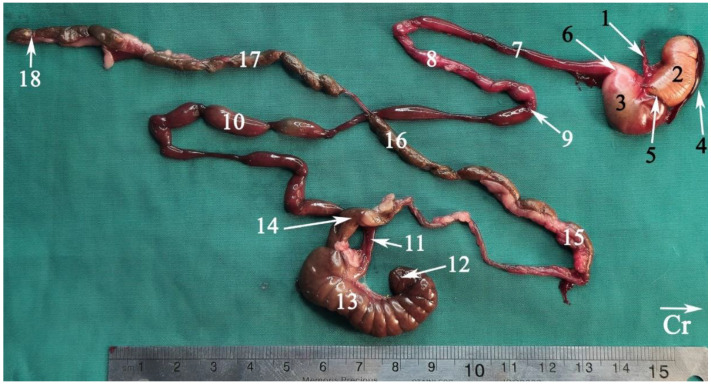
Gastrointestinal tract of a fresh cadaver of a male Syrian hamster *ex situ*: right view. (1) Esophagus, (2) non-glandular part of the stomach, (3) glandular part of the stomach, (4) spleen, (5) gastric groove, (6) pylorus, (7) descending duodenum, (8) ascending duodenum, (9) duodenal–jejunal flexure, (10) jejunum, (11) ileum, (12) cecal apex, (13) cecal body, (14) colon ampulla, (15) ascending colon, (16) transverse colon, (17) descending colon, (18) rectum. Cr: cranial.

**Figure 6:**
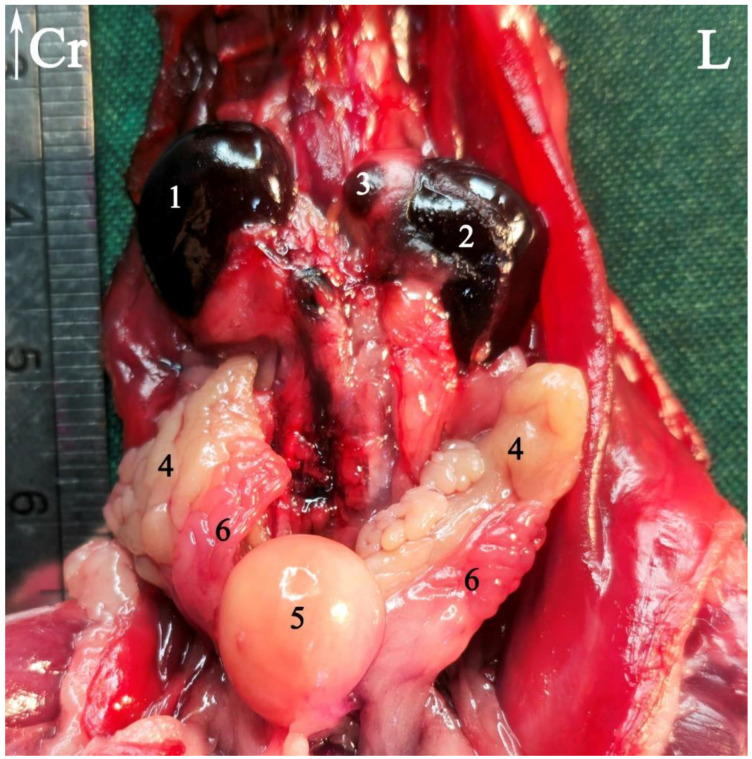
Some arrangements of the abdominal urogenital system of a male Syrian hamster *in situ*: ventral view. The left seminal vesicle and coagulating gland are displaced laterally. (1) Right kidney, (2) left kidney, (3) left adrenal gland, (4) seminal vesicle gland, (5) bladder, (6) coagulating gland. Cr: cranial, L: left side of the body.

**Figure 7:**
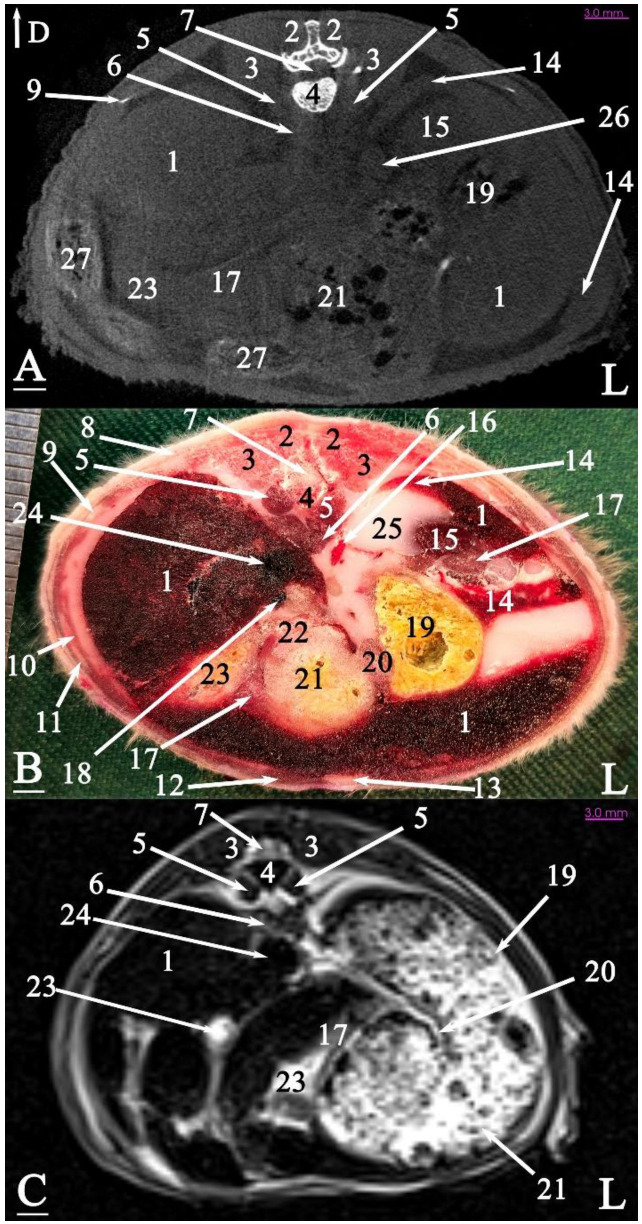
Transverse slice of micro-CT (**A**), cadaver (**B**), and MRI (**C**) of a male Syrian hamster abdomen, corresponding to line 1 in Figure 1: cranial aspect. (1) Liver, (2) lumbar multifidus muscle, (3) erector spinae muscle, (4) second lumbar vertebra, (5) quadratus lumborum muscle, (6) diaphragm crura, (7) spinal cord, (8) internal abdominal oblique muscle, (9) rib, (10) transversus abdominis oblique muscle, (11) external abdominal oblique muscle, (12) rectus abdominis muscle, (13) line alba, (14) spleen, (15) left kidney, (16) aorta, (17) pancreas, (18) cranial mesenteric vein, (19) non-glandular part of the stomach, (20) gastric groove, (21) glandular part of the stomach, (22) pylorus, (23) duodenum, (24) caudal vena cava, (25) adipose tissue, (26) adrenal gland, (27) ascending colon. D: dorsal, L: left side of the body (scale = 3 mm in (**A**,**C**)).

**Figure 8:**
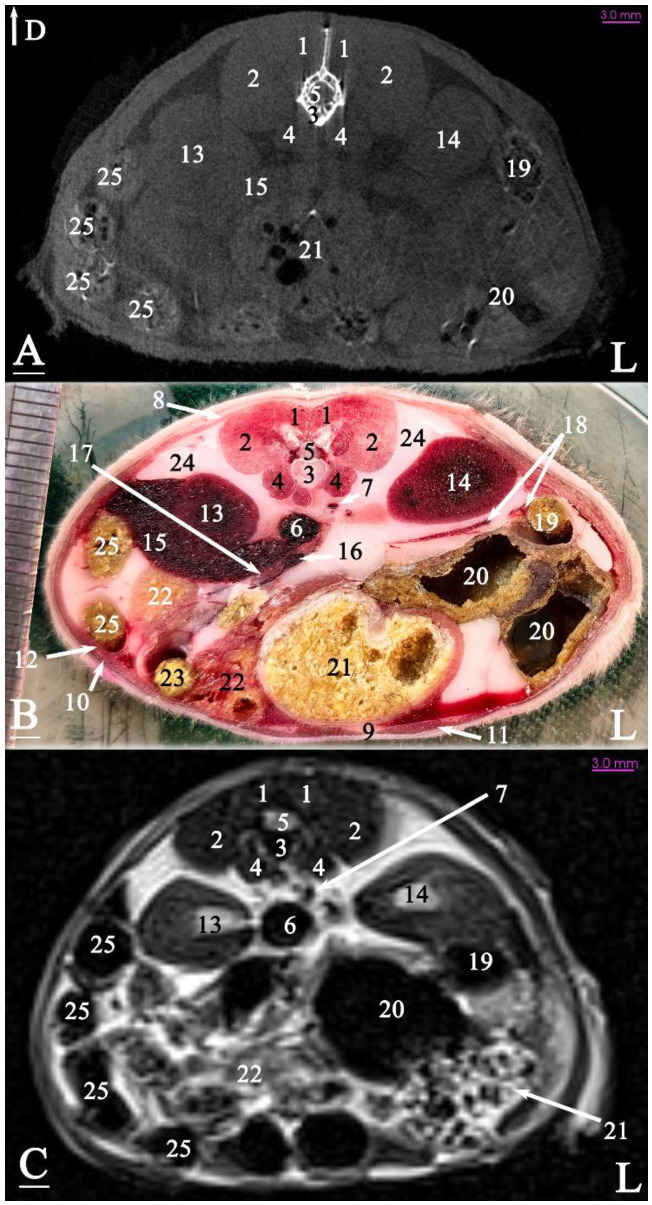
Transverse slice of micro-CT (**A**), cadaver (**B**), and MRI (**C**) of a male Syrian hamster abdomen, corresponding to line 2 in Figure 1: cranial aspect. (1) Lumbar multifidus muscle, (2) extensor of the vertebral column, (3) fourth lumbar vertebra, (4) quadratus lumborum muscle, (5) spinal cord, (6) caudal vena cava, (7) aorta, (8) internal abdominal oblique muscle, (9) line alba, (10) external oblique abdominal muscle, (11) rectus abdominal muscle, (12) transversus abdominis oblique muscle, (13) right kidney, (14) left kidney, (15) liver, (16) cranial mesenteric vein, (17) pancreas, (18) spleen, (19) descending colon, (20) cecum, (21) glandular portion of the stomach, (22) jejunum, (23) duodenum, (24) fat, (25) ascending colon. D: dorsal, L: left side of the body (scale = 3 mm in (**A**,**C**)).

**Figure 9:**
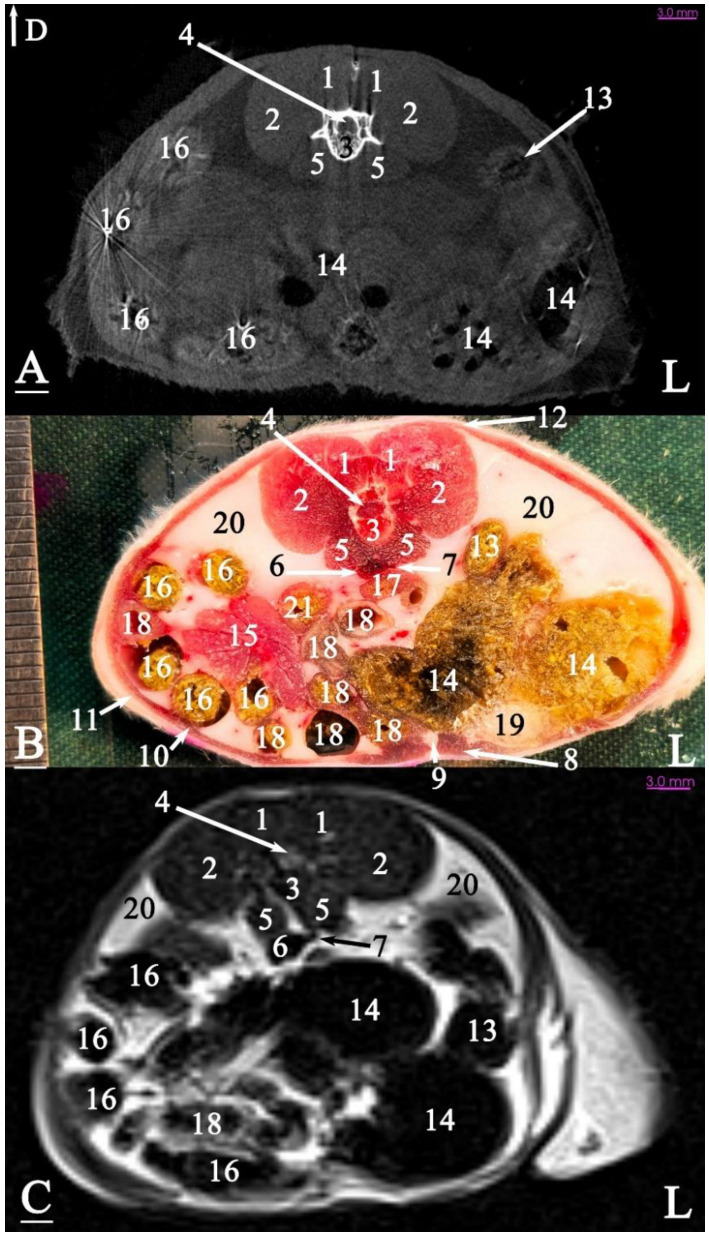
Transverse slice of micro-CT (**A**), cadaver (**B**), and MRI (**C**) of a male Syrian hamster abdomen, corresponding to line 3 in Figure 1: cranial aspect. (1) Lumbar multifidus muscle, (2) erector spinae muscle, (3) sixth lumbar vertebra, (4) spinal cord, (5) psoas muscle, (6) cranial vena cava, (7) aorta, (8) rectus abdominal muscle, (9) line alba, (10) transversus abdominis oblique muscle, (11) external abdominal oblique muscle, (12) internal abdominal oblique muscle, (13) descending colon, (14) cecum, (15) pancreas, (16) ascending colon, (17) ascending duodenum, (18) jejunum, (19) seminal vesicle gland, (20) fat, (21) descending duodenum. D: dorsal, L: left side of the body (scale = 3 mm in (**A**,**C**)).

**Figure 10:**
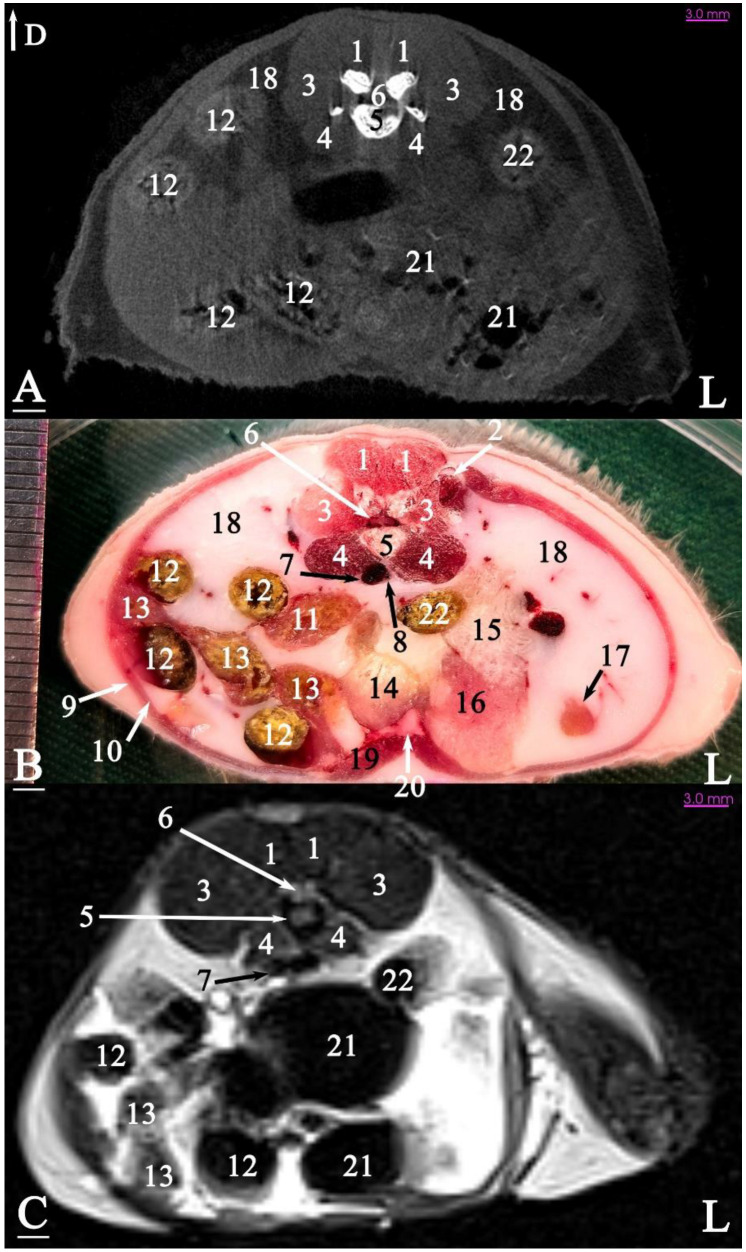
Transverse slice of micro-CT (**A**), cadaver (**B**), and MRI (**C**) of a male Syrian hamster abdomen, corresponding to line 4 in Figure 1: cranial aspect. (1) Lumbar multifidus muscle, (2) middle gluteal muscle, (3) extensor of the vertebral column muscle, (4) psoas muscle, (5) seventh lumbar vertebra, (6) spinal cord, (7) caudal vena cava, (8) aorta, (9) external abdominal oblique muscle, (10) transversus abdominis oblique muscle, (11) retroperitoneal duodenum, (12) ascending colon, (13) ilium, (14) bladder, (15) seminal vesicle gland, (16) coagulating gland, (17) left testicle, (18) fat, (19) rectus abdominis muscle, (20) linea alba, (21) cecum, (22) descending colon. D: dorsal, L: left side of the body (scale = 3 mm in (**A**,**C**)).

**Figure 11:**
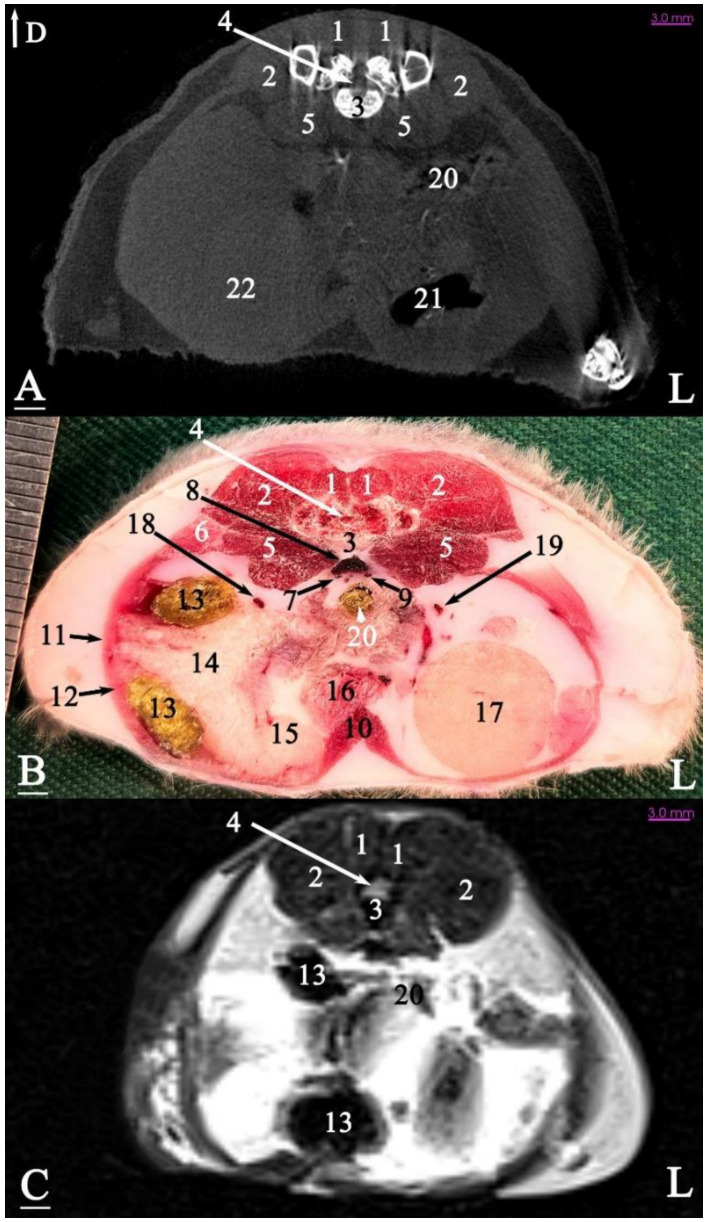
Transverse slice of micro-CT (**A**), cadaver (**B**), and MRI (**C**) of a male Syrian hamster abdomen, corresponding to line 5 in Figure 1: cranial aspect. (1) Lumbar multifidus muscle, (2) middle gluteal muscle, (3) first sacral vertebra, (4) spinal cord, (5) psoas major muscle, (6) tensor fasciae latae muscle, (7) right external iliac vein, (8) caudal vena cava, (9) left external iliac vein, (10) rectus abdominis muscle, (11) external abdominal oblique muscle, (12) transversus abdominis oblique muscle, (13) ascending colon, (14) seminal vesicle gland, (15) coagulating gland, (16) prostate, (17) left testis, (18) right external iliac artery, (19) left external iliac artery, (20) descending colon, (21) cecum, (22) right testicle. D: dorsal, L: left side of the body (scale = 3 mm in (**A**,**C**)).

**Figure 12:**
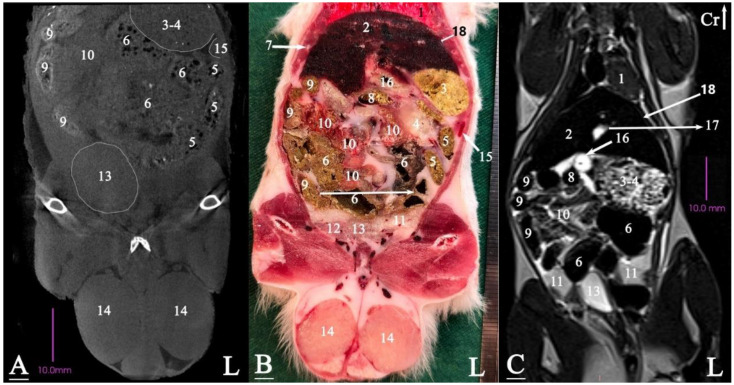
Dorsal slice of micro-CT (**A**), cadaver (**B**), and MRI (**C**) of a male Syrian hamster abdomen, corresponding to line 6 in Figure 1: Dorsal segment. (1) Lungs, (2) liver, (3) non-glandular part of the stomach, (4) glandular part of the stomach, (5) descending colon, (6) cecum, (7) rib, (8) transverse colon, (9) ascending colon, (10) ileum, (11) seminal vesicle gland, (12) coagulating gland, (13) bladder, (14) testis, (15) spleen, (16) duodenum, (17) gall bladder; (18) diaphragm. Cr: cranial, L: left side of the body (scale = 10 mm in (**A**,**C**)).

**Figure 13:**
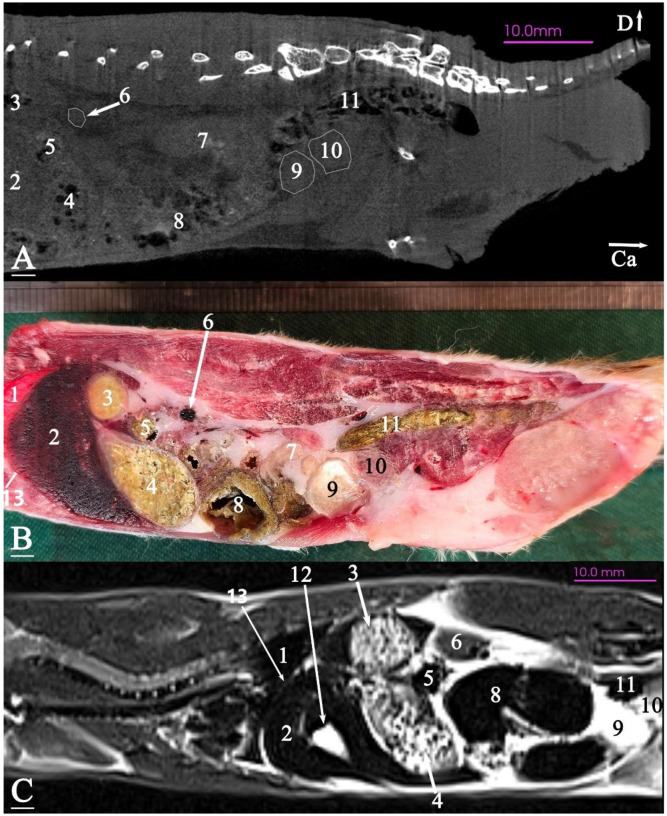
Sagittal slice of micro-CT (**A**), cadaver (**B**), and MRI (**C**) of a male Syrian hamster abdomen, corresponding to line 7 in Figure 1: left lateral view. (1) Lung, (2) liver, (3) non-glandular part of the stomach, (4) glandular part of the stomach, (5) transverse colon, (6) left kidney, (7) ilium, (8) cecum, (9) bladder, (10) prostate, (11) descending colon, (12) gall bladder; (13) diaphragm. Ca: caudal, D: dorsal (scale = 10 mm in (**A**,**C**)).

In radiograph projection, yellow lines were used to indicate the dispositions of the transverse, dorsal, and sagittal slices (Figure 1). The abdominal topographic anatomy of the Syrian hamster is illustrated from the right and left lateral and ventral views (Figure 2, Figure 3 and Figure 4). The arrangement of the gastrointestinal tract with the urinary and reproductive systems *in situ* or without them in *ex situ* is displayed (Figure 2, Figure 3, Figure 4, Figure 5 and Figure 6). The preliminary examination, clinically relevant arrangements of the Syrian hamster’s abdomen were not identified using 1.5 Tesla magnetic resonance imaging (MRI). Six transverse cadaver slices (Figure 7, Figure 8, Figure 9, Figure 10 and Figure 11), one dorsal cadaver slice (Figure 12), and one sagittal cadaver slice (Figure 13) were compared with their corresponding micro-CT and MRI images. Cranial views of the transverse slices are provided (Figure 7, Figure 8, Figure 9, Figure 10 and Figure 11). The dorsal slice was obtained at the level of the mid-femur region of the body (Figure 12), and the sagittal slice was taken laterally to the lumbar transverse processes (Figure 13). No abnormalities or diseases were detected in the abdominal region of the hamsters. 

### 3.1. Topographic Anatomy

The topographical anatomy of the digestive and urogenital systems in the middle and caudal regions of the Syrian hamsters was as follows: The liver was divided into four lobes: the right dorsal–caudal lobe, left dorsal–caudal lobe, dorsal medial lobe, and ventral medial lobe (Figure 2, Figure 3 and Figure 4). Most of the liver was located in the cranial part of the abdomen. The stomach was divided into two regions: the glandular portion was situated along the midline, and the non-glandular portion was located on the left side of the abdomen (Figure 2, Figure 4 and Figure 5). The pancreas consisted of three lobes: the gastric lobe, connected to the stomach and pylorus; the splenic lobe, located caudal to the spleen (Figure 2 and Figure 4); and the duodenal lobe, which was identified as the smallest. The spleen, which was shaped like the letter “L”, was located on the left side of the abdomen. The small intestine was composed of three segments: the duodenum, jejunum, and ileum. The duodenum was configured in a U-shape, consisting of the descending duodenum on the right and the ascending duodenum on the left side of the abdomen (Figure 5). The jejunum, transitioned from the pylorus, featured numerous small loops and was positioned on the right side of the abdomen (Figure 2, Figure 3, Figure 4 and Figure 5). The relatively short ileum was connected to the jejunum and the cecum (Figure 5). The large intestine included the cecum, ascending colon, transverse colon, and descending colon. The cecum, the most prominent dilation of the intestinal tract, was described as sac-like in appearance and divided into the apex (on the median line) and the body (on the left side), lacking a cecal appendix and having tenia (Figure 2, Figure 3, Figure 4 and Figure 5). The ascending colon formed a U-shaped loop on the right side of the abdomen (Figure 3, Figure 4 and Figure 5). In contrast, the transverse colon was extended horizontally from the right side to the left, across the caudal part of the abdomen (Figure 5). The descending colon ran along the left side of the abdomen, leading to the rectum and anus (Figure 5). The kidneys, light brown and bean-shaped, were positioned along the midline in the lumbar position, with the right kidney being slightly more cranial than the left (Figure 2, Figure 3 and Figure 6). At the level of the cranial extremity of each kidney, the arrangements on either side of the caudal vena cava were the adrenal glands (Figure 6). The ureters and prostate were not readily visible. The bladder was located in the midline of the caudal part of the abdomen (Figure 4 and Figure 6). The vesicular glands (the large, lobulated yellow arrangements) and the darker-colored, coagulating glands (closely related to the concave surface of the vesicular glands) were situated on both sides of the mid-caudal abdomen (Figure 2, Figure 3, Figure 4 and Figure 6).

### 3.2. Anatomical Sectioning

#### 3.2.1. Transverse Cadaver Slices

The slice in Figure 7 was made at the level of the second lumbar vertebra and represents the last disposition where the diaphragm crura were visible. The liver was positioned at the ventral surface of the abdominal cavity, located on the right side, adjacent to the right body wall. A considerable part of the remaining abdominal cavity was occupied by the stomach. The caudal vena cava was positioned slightly to the right and ventrally, surrounded by the liver. A small part of the pancreas was observed ventral to the left kidney, caudal to the stomach, and another portion was seen at the junction of the duodenum and the stomach. The spleen was located on the left side of the midline, between the abdominal wall and the stomach, and was clearly visible in this slice. Additionally, the left kidney was situated between the liver and the stomach.

The slice in Figure 8 was made at the level of the fourth lumbar vertebra, as the last slice where the liver was visible (at the level of the right kidney). The stomach was located in the midline and floor of the abdominal cavity. The most caudal part of the spleen was observed in this slice. All small intestines were visible on the right side of the abdomen. The left kidney was positioned adjacent to the left abdominal wall. This was the first slice where the cecum was observed, positioned on the left side of the midline next to the abdominal wall, while the descending colon was located adjacent to both the left abdominal wall and the cecum. The other intestinal masses were located in the lower right side of the abdominal cavity.

The slice in Figure 9 was made at the level of the sixth lumbar vertebra, showing that both kidneys were terminated at this level. The cecum occupied a significant portion of the left half of the abdominal cavity. The ascending duodenum was first observed in this slice, located on the ventral aspect of the sublumbar muscles. A large portion of the pancreas was visible in this slice, positioned on the right side of the midline among the intestinal mass. A portion of the left seminal vesicle gland was observed on the ventral aspect of the cecum, adjacent to the ventral surface of the abdominal cavity. The cranial vena cava was immediately located on the ventral aspect of the lumbar vertebral column.

The slice in Figure 10 was made at the level of the seventh lumbar vertebra. Most of the left half of the abdominal cavity was filled with fat. For the first time, a small portion of the testicle was embedded within this fat. The bladder was located almost in a sagittal position at the ventral surface of the abdominal cavity. The right half of the abdominal cavity was occupied by the intestinal mass (except the descending colon) and fat. The seminal vesicles and coagulating glands were positioned nearly in the sagittal plane on the left side of the midline.

The slice in Figure 11 was made at the level of the first sacral vertebra. In the midline, the caudal vena cava and the descending colon were visible at the abdominal level. Most of the right half of the abdominal cavity was occupied by the seminal vesicle and the coagulating gland. The left half of the abdominal cavity was covered by the left testis and adipose tissue. Thus, the intestinal mass was not observed on the left side of the abdominal cavity for the first time in this slice. The prostate was located in the midline at the bottom of the abdominal cavity.

#### 3.2.2. Dorsal Cadaver Slice

The stomach was located on the left side of the body and caudal to the liver. Most of the small intestinal mass was positioned on the right side and midline of the abdominal cavity, predominantly connected to the cecum on the right side. The paired seminal vesicles and coagulating glands were situated on either side of the bladder in the caudal part of the abdominal cavity. The testes were located outside the abdominal cavity in the scrotum (Figure 12).

#### 3.2.3. Sagittal Cadaver Slice

The stomach was positioned entirely caudal to the liver, and the transverse colon was situated between the glandular and non-glandular parts of the stomach. The glandular portion of the stomach was in contact with the ventral surface of the abdomen, while the non-glandular part was positioned on the ventral surface of the lumbar vertebrae. The small intestine was located on the ventral surface of the sublumbar muscles in the middle and caudal regions of the abdomen, surrounded by adipose tissue. The cecum was located on the abdominal floor, in contact with the stomach cranially and the bladder caudally. The descending colon and rectum were located on the ventral surface of the caudal and sacral vertebrae. The prostate was situated caudal and dorsal to the bladder, almost at the level of the second sacral vertebra (Figure 13).

### 3.3. Micro-CT Slices

In the micro-CT imaging of the Syrian hamster’s abdomen, bony arrangements such as vertebrae and ribs were clearly visible as hyperdense areas, while the spinal cord appeared isodense. Subcutaneous tissue and fat were shown as hypodense, and the skin, abdominal wall muscles, epaxial muscles, and sublumbar muscles appeared as isodense (Figure 7, Figure 8, Figure 9, Figure 10, Figure 11, Figure 12 and Figure 13). The diaphragm crura, located among adipose tissue, were seen as an isointense structure (Figure 7). The absence of contrast media made it challenging to delineate veins and arteries in the gross slices. Liver tissue was observed as isodense (Figure 7 and Figure 8). The gastrointestinal tract exhibited hypodense regions due to air content, with gastric folds not visible. The duodenum was sometimes identified only when emerging from the stomach (Figure 7), and the small intestine was not easily distinguished from the large intestine due to minimal air content, with only approximate topographic boundaries observable. The stomach, cecum, descending colon, transverse colon, and ascending colon were seen as hyperdense, with well-differentiated margins (Figure 7, Figure 8, Figure 9, Figure 10, Figure 11, Figure 12 and Figure 13).

The spleen was positioned ventral and cranial to the kidney, appearing isodense relative to the kidney and hypodense relative to the liver (Figure 7). The pancreas appeared isodense to the spleen and hypodense to the liver (Figure 7). The kidneys were isodense (Figure 8), and the adrenal glands were isodense relative to the kidneys (Figure 7). The bladder and prostate were isodense in the midline of the body (Figure 12 and Figure 13). The gallbladder, intervertebral disc, ureter, and accessory sex glands, including the seminal vesicle and coagulating glands, were difficult to identify.

### 3.4. MRI Slices

In this study, both T1-weighted (T1W) and T2-weighted (T2W) sequences of the animal’s body were acquired and examined. Due to the poor visibility of most abdominal arrangements in nearly all T1W sequences, T2W images were selected and used for further analysis. The following image demonstrates both sequences and explains the preference for T2W imaging for the abdominal cavity in this research. No structure within the abdominal cavity was observed to be devoid of signal.

The bony arrangements, including the lumbar vertebrae and ribs, were clearly visible and hypointense in the MRI images. The spinal cord appeared hyperintense, rendered in white against the black background of the vertebrae (Figure 7, Figure 8, Figure 9, Figure 10, Figure 11, Figure 12 and Figure 13). The central part of the intervertebral disc of the lumbar vertebrae appeared hyperintense, while the peripheral fibrous part was seen as hypointense (Figure 10). The skin, subcutaneous tissue, and fat showed bright signals (hyperintense), making them easily identifiable due to their contrast with adjacent anatomical arrangements (Figure 7, Figure 8, Figure 9, Figure 10, Figure 11, Figure 12 and Figure 13). Muscles, such as those of the abdominal wall, epaxial muscles, and sublumbar muscles, exhibited intermediate signals (isointense) and were seen in grey. Differentiating the abdominal wall muscles was almost impossible due to their uniform isointensity. The diaphragm crura, located among adipose tissue, were seen as an isointense structure (Figure 7). Blood vessels, including the caudal vena cava and aorta, were discerned by their low signal intensity (hypointense) and anatomical dispositions, appearing almost circular in transverse slices (Figure 7, Figure 8, Figure 9, Figure 10, Figure 11 and Figure 13). The gallbladder was shown as highly hyperintense within the hypointense liver tissue (Figure 12 and Figure 13).

The normal presence of air within the gastrointestinal tract was associated with some or all regions of these structures being observed as hypointense. However, because of the minimal air in the initial part of the tract and the presence of undigested food, the small intestine (except the duodenum) appeared hyperintense relative to the liver, while the stomach and duodenum were observed as very hyperintense (Figure 7). The large intestine was observed as isointense compared to the caudal vena cava. The differentiation between the stomach and the cecum (completely hypointense) was more evident in this imaging modality than in CT imaging (Figure 8). The pancreas was seen between the duodenum and stomach and appeared hypointense compared to the duodenum (Figure 7). The kidneys were highly hyperintense compared to the liver, with the renal pelvis appearing hyperintense due to the presence of urine. The bladder and prostate, likely not visible in transverse slices, were observed as hyperintense in the sagittal and dorsal slices (Figure 12 and Figure 13). The seminal vesicle and coagulating glands were only visible in the dorsal slice and hardly detectable in the transverse slices. It is also hard to differentiate these two glands. They appeared as hyperintense (Figure 12). The spleen, ureter, and adrenal glands were challenging to identify.

## 4. Discussion

Due to the inherently small size of Syrian hamsters, the clinically relevant anatomical structures of the abdomen present challenges for visualization using conventional CT. However, micro-CT provides clear delineation of almost all abdominal organs, demonstrating its potential as a valuable tool for clinical diagnosis. Compared with helical CT, the promising advantages of MCT include high bone sensitivity [10]. However, similar to conventional CT, there is relatively poor contrast of soft tissue in micro-CT [62]. Based on our findings, micro-CT without contrast demonstrates superior utility in identifying bones and foreign objects, while MRI proves more effective in locating the nervous system (specifically the spinal cord), lumbar intervertebral disks [63], abdominal aorta, and caudal vena cava. On the other hand, spinal fractures can occur from trauma in any small mammals [64], and CT cannot be used to detect small orthopedic lesions in smaller companion animal species such as rodents and rabbits; however, MCT is suitable for the evaluation of small bone fractures in any anatomic region [65]. Micro-CT clearly provides detailed anatomical information for examining the urinary tract in small mammals [53,66] and the liver and its lesions in rodents [67].

MRI devices used in veterinary medicine typically operate within a range of 0.2 to 3 Tesla. While lower-power MRIs are more affordable and more straightforward to operate, they produce lower-resolution images, longer scan times, increased anesthesia requirements, and a higher risk of motion artifacts. Research has demonstrated that 3 Tesla MRIs provide significantly better image resolution than 1.5 Tesla machines, even in larger animals such as canines, specifically for imaging adrenal glands [68]. For smaller animals, including rodents and rabbits, the enhanced resolution of 3 Tesla MRIs is especially valuable for detailed imaging. Although some studies have explored using 9.4 Tesla MRI for abdominal scans in smaller animals like mice [69], our findings with the more accessible 3 Tesla MRI in Syrian hamsters visualized clinically relevant abdominal anatomic arrangements. This makes the 3 Tesla MRI the recommended option for improving diagnostic accuracy in these species without the need for ultra-high-field systems.

Considering the small size of Syrian hamsters, this study obtained images using a 3 Tesla MRI in both T1 and T2 sequences across transverse, sagittal, and dorsal planes, with the T2 sequence demonstrating superior quality in all slices. The T2 sequence provided better visualization of the most clinically significant abdominal arrangements compared to the T1 sequence [69], while the liver was best visualized in the T1 sequence [70]. Similarly, the normal anatomy of the abdomen in small animals is best visualized in the T1 and T2 sequences, and 1 Tesla MRI with these sequences has been used in transverse, sagittal, and dorsal planes to evaluate the normal abdomen of dogs and cats [71,72]. 

Although the gross cross-section of the examined hamsters shows less correspondence with their MRI images than the micro-CT images, limited existing data regarding abdominal MRI in common laboratory rodents (CLRs), including Syrian hamsters, rats, mice, guinea pigs, and chinchillas, as well as rabbits, are insufficient for comparative analysis with our findings. On the other hand, both imaging techniques exhibited remarkable accuracy in depicting the gastrointestinal tract and certain portions of the urinary tract, including the kidneys and bladder. Nevertheless, some structures are better defined in micro-CT or MRI images. The rabbit’s cecum and stomach contain an amount of ingesta and gas [8]. The stomach is better visualized than the cecum in MRI due to fundamental differences in the tissue composition and magnetic properties of these two organs. The stomach is filled with the amount of ingesta, which is rich in water and responds well to MRI signals. The cecum, on the other hand, is mostly filled with air and has less soft tissue density, as is typical of animals that are foregut fermenters. Since air produces very weak signals in MRI (due to the lack of hydrogen atoms, which MRI relies on for imaging), the cecum generates weak signals. Gas can be used as a gastrointestinal contrast medium, but it provides only transitory contrast [72]. In conclusion, MRI images provide significantly more distinct differentiation between the cecum and stomach than micro-CT. Moreover, MRI has been employed in the diagnosis of atherosclerosis [73] and the evaluation of stented aortas in the abdominal region of rabbits [74].

Several vessels observed in the gross anatomical section could not be visualized in the corresponding MR and/or micro-CT images due to insufficient contrast resolution, similar to that in rabbits [22], cats [23,71], and dogs [24]. To recognize abdominal vascular structures and facilitate the localization and identification of different visceral anatomical structures of the abdomen in Syrian hamsters, vascular tomographic imaging, including CT and MR angiography, would be a useful technique, similar to studies reported in rabbits [29], cats [72,75,76], and dogs [77]. Furthermore, the use of intravenous contrast CT improved the diagnostic confidence of liver lobe torsion in rabbits [78] and small intestinal obstruction in rabbits [79]. On the other hand, in the paper ’Advances in Micro-CT Imaging of Small Animals’, the advantages and disadvantages of using contrast agents in micro-CT imaging are discussed. The authors mention that due to the inherent lack of contrast for soft tissue imaging, most micro-CT scans rely on high atomic number contrast agents. However, they also state that the use of these agents can present challenges. For example, the use of clinical contrast agents in in vivo small-animal imaging is especially challenging. Due to the significantly higher renal clearance rates in small animals compared to humans, injected contrast agents are rapidly eliminated from their bodies [80]. Accordingly, intravenous administration of contrast medium was not employed in this study, as the primary aim was to document the baseline micro-CT and MRI images acquired through standard procedures.

Pre-imaging fasting reduces gas within the gastrointestinal tract of dogs and cats [2]. Most rodents and rabbits, similar to horses, are hindgut fermenters, with microbial fermentation occurring primarily in the large intestine. However, hamsters are categorized as foregut fermenters, resembling ruminants [81]. Fasting was not implemented in this study. This decision was based on the fact that hamsters, like other rodents and rabbits, are incapable of vomiting [64].

Additionally, due to the small body size and high metabolic rate of laboratory animals, fasting can lead to hypoglycemia and dehydration, potentially within just six hours of food deprivation [82]. In support of this, the rabbit’s stomach is rarely found empty; instead, it is usually observed to be partially filled or full [83]. In this study, the stomachs of the hamsters were observed to be partially filled. Due to the inability of rabbits, guinea pigs, and chinchillas to vomit, gastric dilation is frequently observed, which can be identified using various imaging modalities [84].

In the textbooks and atlases concerning the anatomy of CLRs and rabbits, the primary accessible sources for anatomical slices are Barone et al. [83] in the *Anatomie Comparée de Mammifères Domestiques* and Barone et al. [85] in the *Atlas of Rabbit Anatomy*. These books provide schematic illustrations of the abdominal slices in formalin-fixed rabbits, including four transverse slices, one sagittal slice, and three dorsal slices of the middle and caudal region of the abdomen. Zotti et al. [22] further contributed to this field by presenting four transverse abdominal slices in frozen rabbits, each with a thickness of 1 cm, without providing the sagittal and dorsal images. Nevertheless, in this investigation, for the first time, colorful frozen anatomical slices of the Syrian hamster’s abdomen with a thickness of approximately 5 mm were obtained. It is important to note that the thickness of contiguous transverse slices may hinder the accurate identification of the relations among various abdominal arrangements and their positioning relative to the body’s surface, especially considering the small size of the animal.

One of the most commonly used slices is the transverse plane, which effectively and easily determines the dispositions and relations of the various abdominal arrangements relative to each other and the abdominal wall. This plane is particularly suitable for abdominal diagnostic purposes. For less experienced clinicians, the abdominal sagittal plane is helpful for quickly understanding the topography of the abdominal arrangements in the transverse plane. Moreover, using the dorsal plane can help identify the degree of filling and displacement of the arrangements toward the right and left sides of the abdominal cavity [8]. Ultimately, employing three planes (transverse, sagittal, and dorsal) provides a comprehensive description of the abdominal arrangements of Syrian hamsters.

Among the available literature on the morphology of the abdominal cavity arrangements in CLRs, including Syrian hamsters [38,59,60], rats [38,59,60,84,86] mice [38,59,60], guinea pigs [38,59,60,87,88], and chinchillas [43,89,90,91,92], as well as rabbits [38,59,60,83,85,93,94], the only accessible sources for anatomical slices are Barone et al. [83,85]. Due to the lack of investigations on the sectional anatomy and imaging of the abdomen in rodents, our findings were also compared with photographs and descriptions from the available publications on CT/MRI with or without sectional anatomy of the rabbit abdomen (see Introduction: Paragraph (6) and the above-mentioned references). Consequently, important comparative morpho-topographical differences and similarities in the mid-caudal abdominal organs between the examined hamsters and the aforementioned species were revealed.

### 4.1. Stomach

The stomach morphology of the examined hamsters consists of a two-chambered stomach with both glandular and non-glandular regions, resembling the stomachs of other Myomorpha, like mice, rats, and even rabbits. This contrasts with the single-chambered glandular stomach typical of guinea pigs and chinchillas.

The gastric fundus of rabbits is located mainly on the left of the midline, and the pylorus is in the right cranial abdominal cavity [8]. However, the stomach of the examined hamsters was located in the left cranial part of the abdominal cavity, similar to that in guinea pigs and rats.

Schwarz and Saunders [95], in their book *Veterinary Computed Tomography*, reported that rodent stomachs are predominantly positioned in the right half of the body, which is inconsistent with the present finding.

### 4.2. Intestines

Unlike rabbits [8], all segments of the small and large intestines of the examined hamsters could not be distinguished, and the ileum was not recognizable in the micro-CT and MRI.

### 4.3. Ileum

Unlike the examined hamsters and other CLRs and chinchillas [96], the terminal portion of the ileum in rabbits ends with the rounded sacculus rotundus at the ileocecocolic junction. Such an anatomical characteristic may play a role in the easy identification of the rabbit ileum sectional imaging and is a common site of foreign body impaction due to luminal narrowing [64].

Samii et al. [72] did not visualize the ileum in the transverse MRI and gross sections of cats, similar to the examined Syrian hamsters. The short size of the structure makes identification more difficult.

### 4.4. Jejunum

Like rabbits and CLRs, the jejunum in the examined Syrian hamsters was the longest part of the small intestine.

In the rabbit, the mass of jejunal loops is mainly positioned in the cranial abdomen, mostly on the left side and dorsal to the cecum [8]. In mice, the jejunum fills the mid-ventral abdominal cavity [97]. However, in guinea pigs [64] and rats [86], the jejunum is located on the right side of the abdominal cavity [64], similar to that in the examined hamsters.

### 4.5. Cecum

Like rabbits and other CLRs, the cecum of the examined hamsters was the largest and most prominent organ in the abdominal cavity.

The cecal appendix is commonly observed only in rabbits, and it is typically absent in our examined hamsters and CLRs.

In contrast to rabbits, guinea pigs, dwarf hamsters, chinchillas, and the examined Syrian hamsters, the mouse and rat cecum are not sacculated and do not possess tenia [97].

In rabbits, the cecum is commonly extended from the level of the first (second) lumbar vertebra to the seventh lumbar vertebra or the first sacral lumbar vertebra, and the positional variation is mainly related to gas distention [8]. However, in the transverse three-matched slices of the cecum of the examined hamsters, it was extended from the level of the third lumbar vertebra to the seventh lumbar vertebra.

The position of the cecum varies with the species. The rabbit cecum is so large that it occupies most of the floor and the right abdominal cavity from the middle to the caudal region, whereas the cecum in the examined hamsters and other CLRs is located in the left half of the middle abdominal cavity. Therefore, the rabbit cecum is not extended to the left lateral of the abdominal cavity when compared to all CLRs.

Capello and Lennox [54], in their book *Clinical Radiology of Exotic Companion Mammals*, and Silverman and Tell [25], in their book *Radiology of Rodents, Rabbits and Ferrets*, showed that the cecum in the male Syrian hamster exits on both sides of the abdominal cavity, which contradicts our findings. Such a positional variation may be related to its long mesentery and gas distention. In the rabbit, gas distention may cause positional variation [8]. For dynamic organ systems, such as the gastrointestinal and vascular systems, CT imaging offers valuable anatomical insights into living animals, providing advantages over conventional anatomical dissection [98]. Therefore, we believe that our findings are more reliable than those presented in radiographic images of the abdomen [25].

### 4.6. Ascending Colon

Unlike the examined hamsters and other CLRs, rabbits have ampulla coli as an enlargement of the beginning of the ascending colon (proximal colon). Such an anatomical feature may cause sacculitis and appendicitis in rabbits [99].

Like the dwarf and European hamsters, mice, rats, and guinea pigs, the ascending colon in the studied Syrian hamsters did not exhibit tenia and haustra. Nevertheless, such anatomical features are reported in rabbits and chinchillas [92].

Similar to rabbits [8], chinchillas [92], and dwarf hamsters [100], the ascending colon of the studied hamsters is mainly located on the right side of the abdominal cavity.

### 4.7. Gall Bladder

Although the presence and absence of the gall bladder vary among species, the pear-shaped gall bladder lies in a fossa on the visceral surface of the liver, with which it is firmly united in rabbits, CLRs, and the examined hamsters.

The presence of a gallbladder in the livers of the examined hamsters is consistent with its presence in mice, guinea pigs, chinchillas, and rabbits. However, it is absent in European hamsters and rats.

The gallbladder is typically located on the right side of the abdomen in rabbits, guinea pigs, and chinchillas, though it may extend to the midline. In contrast, in mice and the studied hamsters, it is positioned on the left side of the abdomen, possibly extending toward the midline.

### 4.8. Pancreas

Like rabbits and CLRs, the pancreas of the Syrian hamster is situated transversely on the dorsal part of the abdomen, in close relationship to the duodenum, stomach, and spleen.

The small size of the Syrian hamsters’ pancreas does make identification more difficult on MCT and MRI images; however, the examined Syrian hamsters’ pancreases can be identified utilizing MRI and MRI slices and surrounding structures as landmarks.

After the administration of intravenous iodinated contrast medium, the feline pancreatic tissue CT enhanced uniformly and hyperattenuated to its pre-contrast state, making it easier to identify [75,101].

### 4.9. Spleen

Although the spleens of rabbits, CLRs, and the examined Syrian hamsters differ in shape and relation, they always lie against the left abdominal wall and are usually covered by the ribs, in close relation to the stomach.

Like the examined Syrian hamsters, the spleen of rabbits is not readily visible on non-contrast CT scans [29]. It was reported that the rabbit spleen could be clearly and reliably identified on post-contrast abdominal CT images. Significantly more spleens were identified in the post-contrast series (93%) than in the pre-contrast series (53%). Therefore, the use of CT or MRI angiography would be helpful in clearly recognizing the spleens of Syrian hamsters.

### 4.10. Kidney

As with other CLRs and rabbits, the kidneys lie in the dorsal wall of the abdomen, placed on either side of the spine, and the cranial pole of the right kidney always lies in the renal impression of the liver. However, the kidneys exhibit asymmetry in their position in this species. In contrast to chinchillas [27] and the examined Syrian hamsters, the right kidney of rabbits was extended to the last rib or thoracic vertebra [30].

The kidney’s MRI intensity in the rabbit T2-weighted images [36] and chinchilla T1-weighted images [27] was heterogeneous compared to the peripheral soft tissues, similar to that in the examined Syrian hamsters.

In rabbit MRI images, the descending colon remained ventromedial to the left kidney, but in the studied hamsters, it was only ventral to the left kidney.

Similar to the transverse MRI images of rabbits [36] and chinchillas [27], the right and left kidneys of the examined Syrian hamsters appeared approximately oval and triangular, respectively. In CT images, the right kidney was oval in chinchillas [27] and bean-shaped in rabbits [36], while the left kidney appeared oval in both species. However, in the Syrian hamsters examined, the right kidney was approximately oval, and the left was triangular.

In transverse MRI images of rabbits [36] and chinchillas [27], the left kidney was surrounded by fat tissues, which helped to identify the left kidney better than the right one, similar to our finding.

### 4.11. Adrenal Glands

Like rabbits and other CLRs, the adrenal glands of the examined hamsters were located medial to the cranial aspect of the kidneys.

MRI and post-contrast CT defined the topography of both glands in the chinchilla [27]. Zotti et al. [22] distinguished the adrenal glands in the CT images of rabbits, but the rabbit glands were identified using MRI [102]. However, the adrenal glands of the examined hamsters were visualized only in MCT images.

### 4.12. Ureter

The ureters of rabbits could be identified in the non-contrast CT images [22]. Nevertheless, the rabbit ureters were visualized in pre-contrast CT due to the contrast provided by the retroperitoneal and abdominal fat. However, the gastrointestinal physiology of rabbits can make identification of the middle and caudal regions of the ureters difficult by compression and displacement [30]. It was reported that ureters are better delineated in post-contrast. On the other hand, the ureters of rabbits could be distinguished by MRI urography [36].

### 4.13. Urinary Bladder

Like rabbits and other CLRs, the bladder of the examined Syrian hamsters, whether full or empty, was almost placed directly on the ventral abdominal wall cranial to the pubis.

Like rabbits, the T2-weighted images demonstrated the urinary bladder as a hyperintense structure prominently positioned in the caudoventral aspect of the left abdominal cavity. In the dorsal plane, the ventral region of the bladder exhibited hyperintensity, whereas the bladder neck appeared hypointense. In the sagittal plane, the bladder lumen displayed a relatively hypointense signal in comparison to the hyperintense bladder wall. The lumen exhibited an irregular shape with indistinct margins in the caudal abdominal region.

### 4.14. Male Accessory Genital Glands

The accessory genital glands are grouped around the pelvic urethra and differ significantly from species to species. Although vesicular and prostate glands have been consistently identified in rabbits, CLRs, and the examined hamsters, the coagulating glands are absent in rabbits, chinchillas, and European hamsters. The seminal vesicles are the most developed sex accessory glands in rabbits, CLRs, and examined hamsters.

Regardless of the presence or absence of some of the accessory genital glands, their topography is almost the same in all the above-mentioned species [89].

#### 4.14.1. Vesicular Gland (Seminal Vesicle)

Unlike rabbits, this gland is paired in examined hamsters and other CLRs. However, the gland was positioned dorsal to the bladder and extended into the abdominal cavity. The shape of the glands varies among the above-mentioned species: the shape was anter-like in guinea pigs [64,88], finger-like projections in chinchillas [64], two-parted in rabbits [83], and in the examined hamsters, the paired vesicular glands were lobulated and crescent-shaped.

Feeney et al. [22], in their book *Atlas of Correlative Imaging Anatomy of the Normal Dog/Ultrasound and Computed Tomography*, could not distinguish the vascular glands in all frozen anatomical sections and CT images. The correlative caudal abdomen and/or pelvic CT images with gross sections were not exhibited in the vesicular glands of rabbits [23], cats [21], and dogs [24]. The current study identified the vesicular glands in the anatomical sections and the MRI. However, correlative pelvic post-contrast CT images with or without gross sections visualized the vesicular gland of rabbits with body weights from 2.8 kg to 3.2 kg [31,103,104]. Consequently, IV administration of contrast medium in micro-CT must be used to define the vascular glands in Syrian hamsters.

A pelvic MRI evaluation of rabbits without gross slices exhibited the vesicular gland [34]. Abdominal MRI images with corresponding cadaver sections did not reveal the vesicular glands of cats [72]. On the other hand, the gland was not identified in the abdominal ultrasonographic evaluation in rats and rabbits [105,106]. In our study, the gland was only seen clearly in a dorsal section of the MRI and was hardly seen in the transverse sections. Seminal vesiculitis should be considered a differential diagnosis in male Syrian hamsters with hematuria [107].

#### 4.14.2. Coagulating Glands

Unlike rabbits, chinchillas, and European hamsters, the coagulating glands are present in rats, mice, guinea pigs, and the examined hamsters. The paired glands are placed on the ventromedial seminal vesicle in rats or the ventrolateral of the seminal vesicle in guinea pigs. They are closely associated with the seminal vesicles and caudal to the bladder.

The shape of the glands varies among the available species. The shape is elongated in rats [86], pyramidal and lobulated in guinea pigs, and lobulated and ellipsoid in the examined hamsters.

No reports have been made on the CT and/or MRI of the glands in the above-mentioned species. However, the glands were not visualized in the abdominal ultrasonographic examination of guinea pigs [108] and rats [106], and the authors did not explain the observations. The coagulating glands were clearly identified in the anatomical sections in the current study. Due to the attachment of the coagulating glands to the vesicular glands, we could not differentiate them in MRI images, and IV administration of contrast medium MCT must be used to define the glands in Syrian hamsters.

#### 4.14.3. Prostate Gland

Although the gland is always present in rabbits and CLRs and is located at the junction of the bladder and urethra, caudal to the coagulating glands, the shape of the glands varies among the available species. The shape of the gland is multi-lobed in the examined hamsters, bi-lobed in guinea pigs and rats, and three-part in rabbits.

The glandular complex of prostate rabbits was entirely visualized within the pelvic cavity by post-contrast CT or MRI scanning of the pelvic region [31,34], while the prostate gland of the examined hamsters was identified in MRI images and the gross section within the pelvic cavity. The IV administration of contrast medium MCT must be used to define the glands in Syrian hamsters.

The intra- and interspecific morphological variations among common rodents and rabbits require careful consideration in clinical practice, particularly concerning diagnostic and treatment options [43,64]. Moreover, a thorough understanding of normal comparative morphology and imaging anatomy of the most commonly used laboratory rodent and rabbit species is essential in biomedical research, as it enables the extrapolation and application of experimental findings to humans [25,38,109]. Consequently, the morphological differences identified in this study should be considered when performing procedures such as gastrostomy or gavage tube placement in hamsters [52,110], cystotomy [111], coeliotomy [112], blood collection from the abdominal caudal vena cava and aorta [113], intraperitoneal injections [43], and blood sampling, as well as the diagnosis and evaluation of hyperadrenocorticism [43,114,115], polycystic disease of the liver and cecal wall [116], intestinal intussusception or prolapse [64], male reproductive tumors [117], and seminal vesiculitis [107] in Syrian hamsters.

## 5. Conclusions

Clinically relevant structures of the examined abdominal regions, identified on transverse, dorsal, and sagittal anatomical sections, were also discerned on the corresponding micro-CT and/or MRI images. The gastrointestinal tract, urinary system, endocrine glands, and large blood vessels, as well as accessory genital glands and musculoskeletal system in the middle and caudal regions of the abdomen, were noted in at least one of the anatomical, micro-CT, and MRI cross-sectional images, regardless of the ileum and ureter. These correlated cross-sectional images reveal the following findings: (1) the presence of a glandular and non-glandular stomach, (2) the stomach and cecum located mainly on the left side of the abdomen, (3) the lack of the ampulla coli, sacculation, and tineae in the ascending colon as well as sacculus rotundus and the cecal appendix, (4) the jejunum located mainly on the right side of the abdomen, (5) the presence of the vesicular, coagulating, and prostate glands, and (6) the right kidney not extending to the area of the last thoracic vertebra. These results resemble those noted in anatomic and radiologic abdomen studies in rats, mice, and guinea pigs, regardless of the rat and mouse sacculated cecum and the guinea pig glandular stomach. However, notable differences were observed compared to those reported in the rabbit abdomen’s sectional anatomy and CT findings. In addition, the presence of the gall bladder and sacculated cecum exhibited interspecific variations among hamsters. The findings from this study provide reference data and an atlas of the normal cross-sectional gross, topographical anatomy photographs, as well as micro-CT and MRI anatomy of the middle and caudal regions of the Syrian hamster abdomen that can be used in the interpretation of any cross-sectional imaging modality.

## Data Availability

All associated data are available in the text.
